# A Systematic Review and Meta-Analysis on the Treatment of Cerebral Hemorrhage with NaoXueShu Oral Liquid

**DOI:** 10.1155/2017/8542576

**Published:** 2017-05-29

**Authors:** Lijun Wu, Yanda Li, Xiaofeng Wang, Xiaomeng Ren, Haiyan Zhu, Yikun Sun, Yanwei Xing, Lingqun Zhu, Yonghong Gao, Hongcai Shang

**Affiliations:** ^1^Key Laboratory of Chinese Internal Medicine of Ministry of Education and Beijing, Key Office of Encephalopathy TCM Research, Dongzhimen Hospital Affiliated to Beijing University of Chinese Medicine, Beijing 100700, China; ^2^Weishi Hospital of Traditional Chinese Medicine, Henan 475500, China; ^3^Beijing University of Chinese Medicine, Beijing 100029, China; ^4^Guang An Men Hospital, China Academy of Chinese Medical Science, Beijing 100053, China

## Abstract

NaoXueShu oral liquid invigorates Qi and promotes blood circulation, which is mainly used for treating the acute stage of the meridian of hemorrhagic apoplexy and acute blood stasis syndrome during early convalescence. Its main clinical manifestations include hemiplegia, mouth askew, hemianesthesia, and inarticulateness. It is used mainly in patients with lobar hemorrhage, basal ganglia, and thalamus of the small amount of bleeding without disturbing consciousness of hypertensive cerebral. The purpose of this study was to evaluate the efficacy and adverse effects of NaoXueShu oral liquid on the treatment of cerebral hemorrhage. In this study, literature on randomized controlled trials was collected from seven databases to evaluate the clinical efficiency of the treatment of cerebral hemorrhage alone or combined with Western medicine. The methodologic quality of the included studies was assessed using a standard Cochrane system review and analyzed using RevMan 5.3.0 software. The study included 14 eligible randomized controlled trials. The results showed that the use of NaoXueShu oral liquid alone or combined with other drugs or auxiliary methods can play a significant role in the treatment of cerebral hemorrhage, especially hypertensive intracerebral hemorrhage.

## 1. Introduction

Intracerebral hemorrhage is a primary nontraumatic hemorrhage, commonly caused by high blood pressure. The mortality rate of patients with cerebral hemorrhage can reach 40% to 50%, and 75% of the survivors cannot live independently after 1 year, which seriously affects quality of life [1]. The main reason for cerebral hemorrhage with high mortality and high disability rates is cerebral edema after brain injury. It affects normal nerve function, increases intracranial pressure, and can form severe cerebral hernia when intracranial pressure increases. Therefore, it is very important to carry out early intervention for patients with cerebral hemorrhage.

NaoXueShu oral liquid's main ingredients include astragalus root, leech, calamus,* Achyranthes*, and* Rhizoma Chuanxiong*; it is intended to replenish Qi and activate blood and remove blood stasis and is mainly used for hemorrhagic stroke in patients with Qi deficiency and blood stasis. It was developed according to the theory that “Qi is the commander of blood; blood is the mother of Qi” [2]. Astragalus is commonly used in Chinese medicine Yiqi, and modern research shows that astragalus can reduce brain damage of neuronal mitochondria after hemorrhage, inhibit neuronal apoptosis, and promote the recovery of neurological function. Moreover, astragalus can also protect the blood-brain barrier permeability, perform antioxidation, and prevent cerebral ischemia [3–5]. Leech has a broken blood and blood stasis-eliminating effect; modern research shows that leech has a cerebral protective effect of anticoagulation, inhibiting platelet aggregation, improving blood rheology, and relieving acute brain injury and brain edema, among other benefits [6–8].

In recent years, experiments have shown that NaoXueShu oral liquid on cerebral vascular disease, especially in the treatment of cerebral hemorrhage, has shown advantages: it can regulate the expression of neuroprotective factor-related protein, reduce the release of inflammatory factors, inhibit free radical damage, inhibit apoptosis, thereby reducing the volume of hematoma, and alleviate cerebral edema. Improving cerebral energy metabolism promotes nerve function repair [9–13].

Modern medicine posits that the key to treating cerebral hemorrhage is to control hypertension, and we are currently studying whether calcium channel blockers, statins, endothelin receptor antagonists, magnesium, erythropoietin, and other drugs can prevent or reverse cerebral hemorrhage; however, evidence is still lacking [14, 15]. In recent years, NaoXueShu oral liquid has been widely used in clinics. Studies found that it can obviously promote the absorption of intracranial hematoma in the treatment of cerebral hemorrhage [16]; promote the recovery of neurological function, especially affecting the treatment of apoplectic aphasia [17–19]; improve blood flow; reduce inflammatory response; effectively reduce brain edema; reduce intracranial pressure; improve the prognosis of patients; reduce the disability rate; and improve the survival rate [20–22].

This study's purpose is to conduct a comprehensive systematic review and evaluate the therapeutic effect of NaoXueShu oral liquid for treating cerebral hemorrhage.

## 2. Methods

This systematic review was conducted in accordance with the guidelines for systematic review and meta-analysis of the preferred reporting items. This systematic study does not require an ethical review.

### 2.1. Databases and Retrieval Strategies

In this study, the Chinese National Knowledge Infrastructure (CNKI), the Chinese Scientific Journal Database, the Chinese Biomedical Literature (CBMdisc), Wanfang database, EMbase, PubMed, and the Cochrane database were searched for original studies and search was conducted in January 2017. The other related research studies were manually retrieved. The following key words were used alone or in combination: “NaoXueShu"; “NaoXueShu injection”; “nxst”; “cerebral hemorrhage”; “hemorrhagic stroke”; “hemorrhagic apoplexy.”

**Figure 1:**
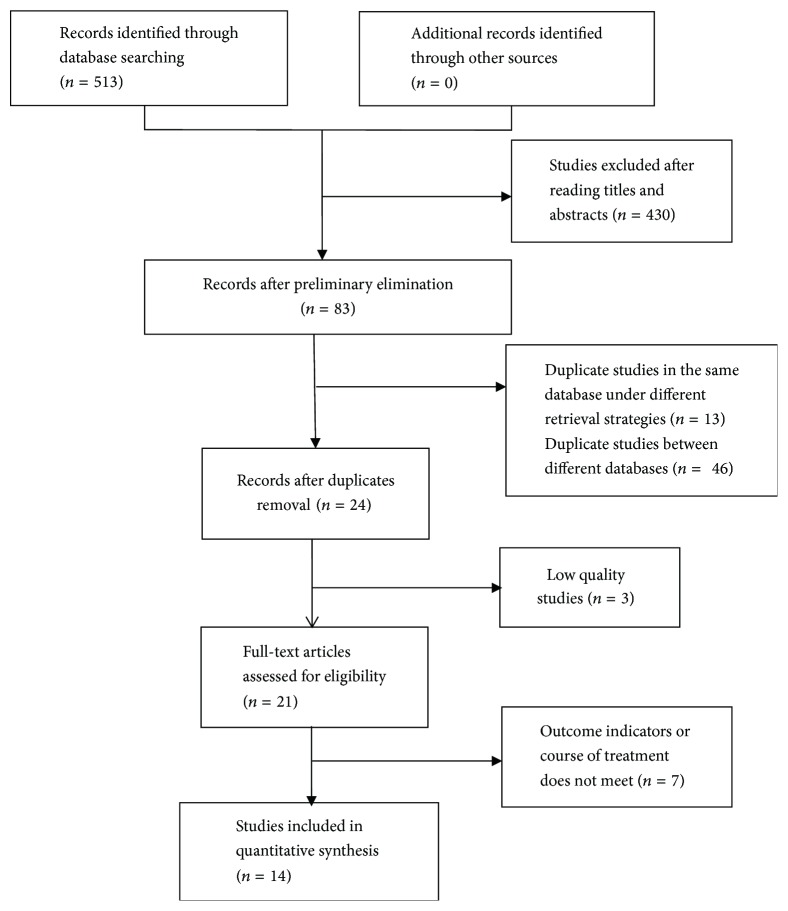
Flow diagram of the systematic review.

More studies were also searched for in the selected references. In addition, we used a flow chart to make the search process more rigorous and detailed (Figure 1).

### 2.2. Inclusion and Exclusion Criteria

#### 2.2.1. Inclusion Criteria

The literature had no special requirements on the language, demographic characteristics, and types of publications. The included patients were not more than 80 years old, and the amount of bleeding was less than 50 mL; moreover, the patients had the basic clinical symptoms of cerebral hemorrhage and, after CT imaging examination, adhered to the clinical diagnostic criteria of cerebral hemorrhage. Oral administration of NaoXueShu oral liquid was 10 mL, oral or nasal feeding, 3 times a day, 1 month as a course of treatment. In the included randomized controlled trials (RCTs), the patients were treated with NaoXueShu alone or in combination with other drugs for treating cerebral hemorrhage. The literature included National Institutes of Health Stroke Scale (NIHSS) score combined with neurologic deficit or corresponding disease diagnostic criteria. The results of the first, second, and third were the efficacy evaluation, NIHSS score at 2 weeks and 4 weeks (in the literature there are descriptive differences of 30 days, 4 weeks, and 1 month; for the convenience of statistics, all of them were unified for 4 weeks), hematoma volume, BI, and GCS.

#### 2.2.2. Exclusion Criteria

We excluded repeat publications in the same group of patients.

### 2.3. Data Screening and Quality Evaluation

**Figure 2:**
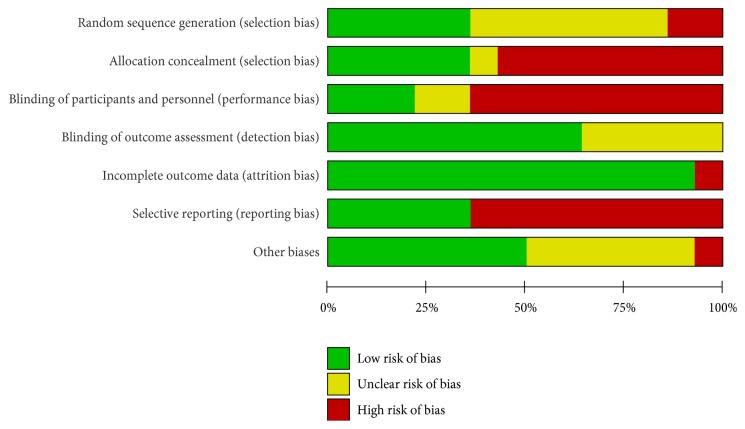
Risk of bias graph. Each item was evaluated as a percentage of the literature, and the quality of the selected literature was evaluated according to the Cochrane criteria.

**Figure 3:**
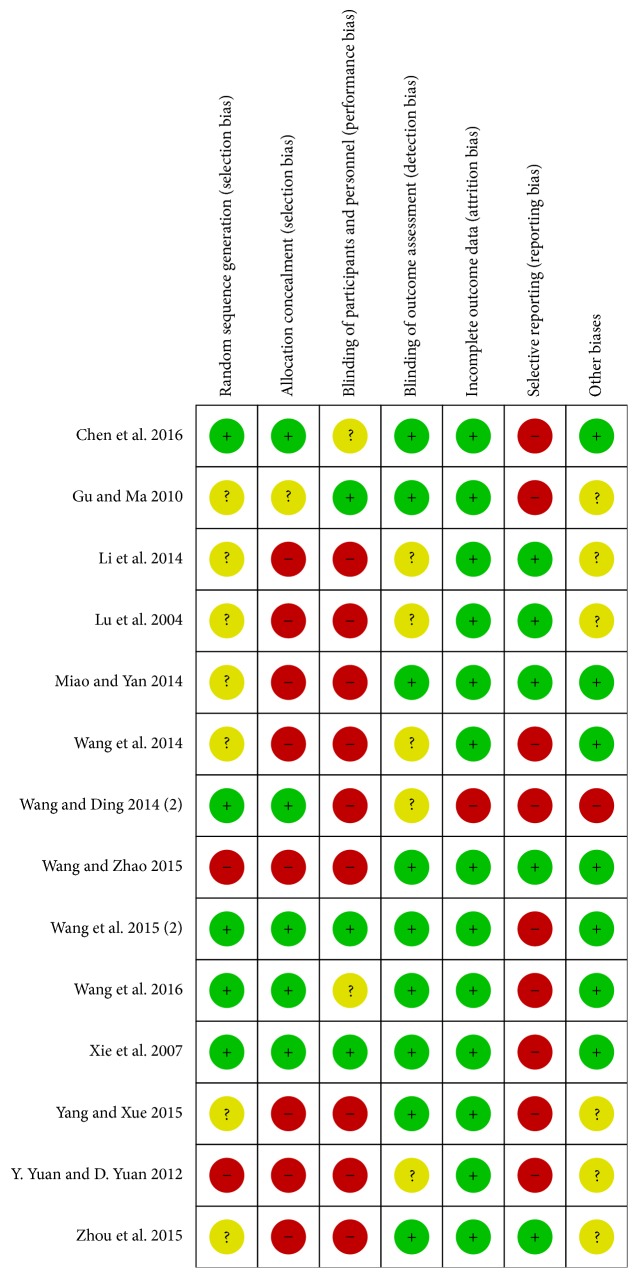
Risk of bias summary: review authors' judgments about each risk of bias item for each included study.

We searched 513 published articles about NaoXueShu treatment of cerebral hemorrhage in 7 databases and excluded the literature that was not consistent and repeated. The remaining 24 articles were screened according to the following criteria: (1) whether the literature focused on patients with cerebral hemorrhage; (2) whether the included samples were randomized controlled studies; (3) whether the final study index of the literature met the requirements; (4) whether the grouping of the literature was scientific; (5) the author's experience of the literature; (6) the published year of the literature. Finally, we got 14 papers. Two authors (Lijun Wu and Xiaofeng Wang) independently performed the literature search, selection, and the data exaction. Any disagreements were discussed, and if the discussion did not yield a final decision, the 3rd author (Xiaomeng Ren) was invited to make a decision. The information included the following: title, author, publication time, literature sources, research scale, number of cases, diagnostic criteria, research methods, and treatment process. We also considered the control group, the results, and adverse reactions. To ensure the quality of the included literature, we used RevMan 5.3.0 software to evaluate the studies systematically and comprehensively. Eventually we obtained the “risk of bias graph” and “risk of bias summary” (Figures 2 and 3). The reliability of the results was further verified by using the forest map to evaluate the efficacy and the funnel plot to evaluate the publication bias.

### 2.4. Statistical Analysis

The RevMan 5.3 software provided by the Cochrane Collaboration was used for data analysis, and, through the study of the content and research indicators for classification, the final packet data entry was carried on. Dichotomous data were expressed as relative risk (RR); continuous outcomes were presented as weighted mean difference (WMD); and the 95% confidence intervals (CIs) were calculated for both. The meta-analysis was performed if the intervention and control groups, as well as the outcomes, were the same or similar. For the significant efficiency, we used Barthel index (BI), the odds ratio (OR) value, hematoma volume, NIHSS score, and Glasgow coma score (GCS), score mean difference (MD) value. According to the results of *I*^2^, we analyzed the data using random (*I*^2^ > 50%, heterogeneity) or fixed effects model (*I*^2^ < 50%).

## 3. Result

A total of 14 studies were incorporated into the information integration (Table 1) [16–18, 20, 21, 23–31].

### 3.1. Characteristics of Included Studies

Initially, we retrieved 513 articles from 7 common databases. At present, NaoXueShu is mainly used in China clinical practice. We collected most of the articles from the Wanfang database and CNKI. In all the articles collected, we excluded duplicate, nonconforming, incomplete data. In addition, according to our requirements, 14 papers were included in the study. All the articles focused on treating cerebral hemorrhage in patients with NaoXueShu.

### 3.2. Methodologic Quality of Included Studies

The selected articles were all screened and belonged to RCT literatures. By choosing 14 articles, we were able to consider a sample size greater than 30 cases. In the 14 articles, 10 were randomized by more scientific methods, including the use of blind method for 3 articles. In the literature, patients in the observation group and the control group had clear inclusion criteria or the patients' data were initially analyzed statistically, if not statistically significant, to be included in the control group. We also objectively analyzed the offset risk, and we evaluated the offset of each article according to the relevant criteria of the offset table. In this process, we tried be objective and fair, especially in the “other biases” column. We were particularly concerned that the 14 articles included the presence of drug promotion of the subjects and made an objective evaluation.

### 3.3. Effects of Interventions

#### 3.3.1. Comparison of Hematoma Volume Change

**Figure 4:**
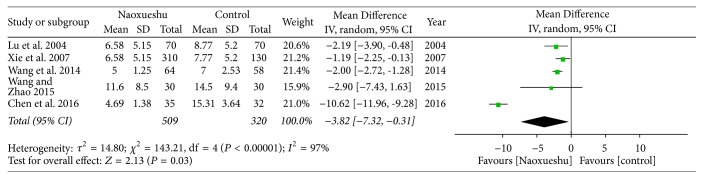
Comparison of hematoma volume.

The following 5 RCTs showed that NaoXueShu can effectively reduce cerebral hemorrhage and had statistical significance (*n* = 509; MD, −3.82; 95% CI, −7.32~−0.31; *I*^2^ = 97%; *P* = 0.03; Figure 4).

#### 3.3.2. GCS Comparison

**Figure 5:**
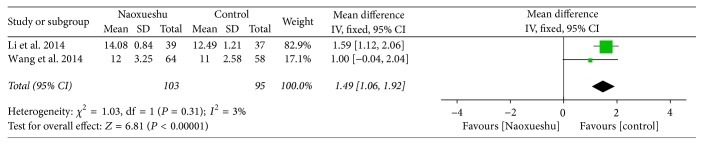
GCS comparison at 2 weeks.

The following 2 RCTs showed that NaoXueShu had a certain effect in treating cerebral hematoma, had obvious function of arousal, and had statistical significance (*n* = 198; OR, 1.49; 95% CI, 1.06~1.92; *I*^2^ = 3%; *P* < 0.00001; Figure 5).

#### 3.3.3. NIHSS Score Comparison

**Figure 6:**
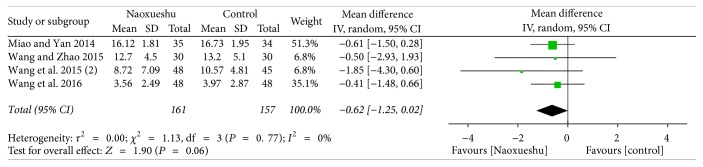
NIHSS score comparison at 2 weeks.

**Figure 7:**
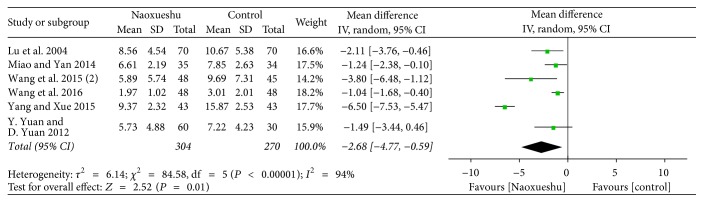
NIHSS score comparison at 4 weeks.

The following 7 RCTs showed NaoXueShu oral liquid's effect on cerebral hemorrhage after 2 weeks and 4 weeks of nerve function protection. The results showed that, 2 weeks after treatment of cerebral hemorrhage, the difference was not statistically significant (*P* > 0.05), indicating that NaoXueShu did not show a significant effect in 2 weeks. But, after 4 weeks of treatment, NaoXueShu can improve nerve function to a certain extent and had statistical significance (*n* = 318; MD; −0.62 95% CI; −1.25~0.02; *I*^2^ = 0%; *P* = 0.06; Figure 6) (*n* = 574; MD; −2.68; 95% CI −4.77~−0.59; *I*^2^ = 94%; *P* = 0.01; Figure 7). In a comparison of 2 and 4 weeks of treatment, after cerebral hemorrhage, *P* value gets smaller and smaller, which showed that the difference between the treatment group and the control group increases.

#### 3.3.4. BI Comparison

**Figure 8:**
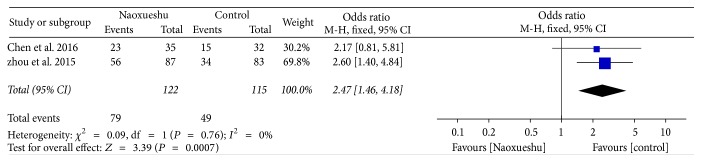
BI comparison at 90 days.

The following 2 RCTs showed that NaoXueShu had a certain effect in treating cerebral hematoma, and it can obviously improve the patient's quality of life and had statistical significance (*n* = 237; OR, 2.47; 95% CI, 1.46~4.18; *I*^2^ = 0%; *P* = 0.0007; Figure 8).

#### 3.3.5. Efficacy Comparison

**Figure 9:**
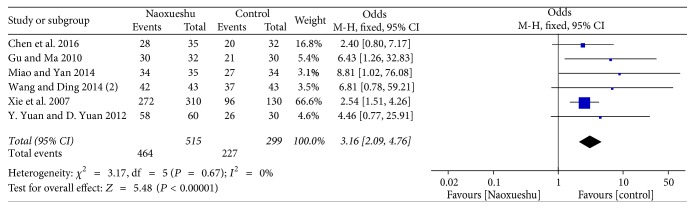
Treatment efficiency comparison.

The following 6 RCTs showed a good effect in treating cerebral hemorrhage compared with the control group and had statistical significance (*n* = 814; OR, 3.16; 95% CI, 2.09~4.76; *I*^2^ = 0%; *P* < 0.00001; Figure 9).

#### 3.3.6. Adverse Reactions

Included in the 14 articles, 2 RCTs were related to adverse reactions, neither of which mentioned the adverse effects of NaoXueShu, which meant this drug had no obvious side effects so far. Therefore, the authenticity and scientific nature of the literature, as well as the integrity of the information provided, have some credibility.

#### 3.3.7. Publication Bias

**Figure 10:**
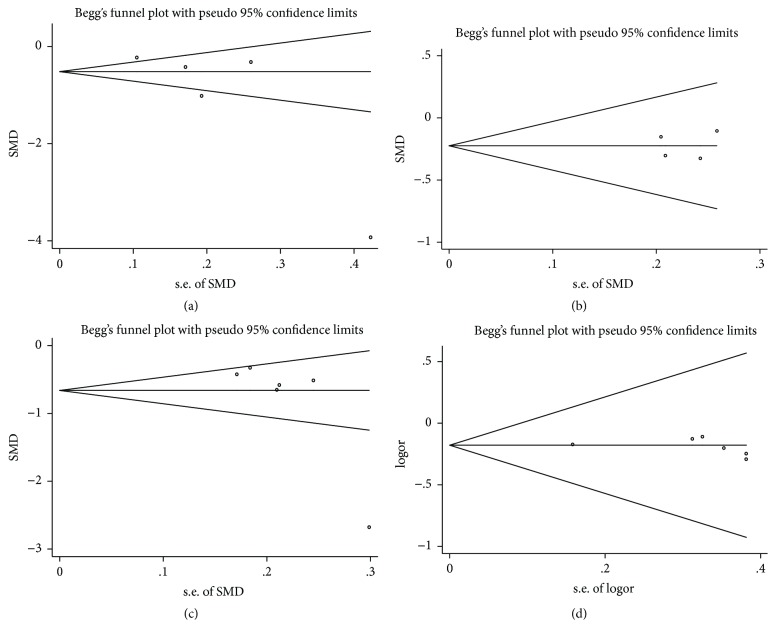
Funnel plots. ((a) Comparison of hematoma volume; (b) NIHSS score comparison at 2 weeks; (c) NIHSS score comparison at 4 weeks; (d) treatment efficiency comparison).

We used Stata software to evaluate the articles' publication bias. Because the indicators of GCS and the BI had only two included RCTs, did not meet the standard of the funnel map, and were without special significance, we conducted the migration analysis to the other 4 indexes and used the Egger method to conduct the diagnosis analysis. The results were shown as in Figure 10 and Table 2.

We used Stata software to conduct migration analysis on the included articles. The results showed that the hematoma volume, NIHSS score at 2 weeks, and efficiency comparison were not offset (*P* > 0.05), while the NIHSS score at 4 weeks shows a slight deviation (*P* < 0.05). Based on this, an NIHSS score at 4 weeks of the literature was removed one by one. We eventually found that when deleting the study by Wang and Zhao [16], the heterogeneity was significantly reduced (from 94% to 19%) and *P* value was increased, indicating that the literature shows an offset in the efficiency comparison.

## 4. Discussion

Cerebral hemorrhage is a common cerebrovascular disease, commonly caused by hypertension. Primitive hematoma first appears after intracerebral hemorrhage, and the hematoma's location and size are closely related to the prognosis, then perihematoma accompanied the emergence of edema. Decreased cerebral blood flow from the perihematoma or peripheral lesion may result in decreased oxygen extraction fraction and tissue ischemic injury, which may be related to the inflammatory process induced by the hematoma [32, 33]. Cerebral hemorrhage and cerebral ischemia belong to the category of “stroke” in the field of Chinese medicine. Chinese medicine's definition of stroke is based on the deficiency of Qi and blood loss because of internal root, emotional disorders, eating Feiganhouwei, or excessive alcohol and tobacco use, causing the imbalance of yin and Yang and reversal of Qi and blood, blood stasis, or blood overflow pulse; therefore, Qi deficiency and blood stasis are the main mechanisms of acute stage and early recovery. NaoXueShu oral liquid's main ingredients include astragalus root, leech, calamus,* Achyranthes*, and* Rhizoma Chuanxiong*, with the effect of tonifying Qi, activating blood, and removing blood stasis. It is suitable for treating cerebral hemorrhage.

NaoXueShu oral liquid is relatively extensive in clinical application. Relevant literature can be found about NaoXueShu, among which the clinical research literature was far greater than experimental studies. This document's purpose is to understand the NaoXueShu's effect of the treatment of cerebral hemorrhage. We divided the literature into five categories: volume of hematoma, GCS, BI, NIHSS score of the patients in each stage (for a period of 2 weeks and 4 weeks), and the curative effect.

There are some advantages and disadvantages of the meta-analysis and the system evaluation: we had a detailed record of the process of selecting the documents. Most included literature was published in Chinese journals. In addition to a small part of the poor quality of literature, most literature was published in more well-known publications with recent publication years. Besides Xie et al. [25] and Lu et al. [27], which were published before 2010, other literatures were published after 2010. In addition, the evaluation system still has many deficiencies: the quality of literature was low, the content was not comprehensive enough (e.g., most of the included RCTs were without follow-up and adverse reactions), and assessment of the neurological function score may be influenced by the subjective factors in the process; therefore, the consistency of the data may differ between documents.

*I* ^2^ is a measure of heterogeneity. The greater *I*^2^, the greater the heterogeneity [34]. According to the statistical results, the heterogeneity of hematoma volume (Figure 4) and NIHSS score at 4 weeks (Figure 7) was 97% and 94%, respectively. The reasons for the high heterogeneity may be related to the number of samples, the quality of the literature, the subject of study, and the duration of treatment. In addition, compared with the two classification variables, high heterogeneity is more common in continuous variables [35], and the volume of hematoma and NIHSS score at 4 weeks were continuous variables, and the possibility of heterogeneity was relatively large. Other GCS of 2 weeks (Figure 5), NIHSS score of 2 weeks (Figure 6), BI of 90 days (Figure 8), and evaluation of the effect (Figure 9) were 3%, 0%, 0%, and 0%, respectively; since they were less than 50%, or even close to 0%, these outcomes cannot have obvious heterogeneity and the results were reliable and relatively stable. However, although there are two indicators of heterogeneity, *P* value of each index is less than 0.05, indicating that the study of NaoXueShu oral liquid in the above indicators is statistically significant.

## 5. Conclusion

NaoXueShu oral liquid is made of BuYangHuanWu decoction from* The Errors in Medicine Corrected* and* Rhubarb Magic Pill from the Golden Chamber*. It is mainly used for removing blood stasis, supplementing tonifying Qi, and activating blood circulation. According to the pathogenesis, “where the bleeding must leave the meridians, blood stasis is left meridian” and the guidelines that state, “Qi is the commander of blood, blood is the mother of Qi, blood gas runs, qi stagnation and blood stasis.” NaoXueShu mainly treated stroke with Qi deficiency and blood stasis. In recent years, it has often been used to treat cerebrovascular disease, and the experimental results show that NaoXueShu oral liquid can obviously improve cerebral microcirculation, inhibit apoptosis, and inhibit oxygen free radicals and protect brain cells function. Clinical study also showed that NaoXueShu oral liquid can reduce the volume of hematoma, improve the recovery of nerve function, reduce brain edema, relieve inflammatory reaction, and promote the prognosis of patients with cerebral hemorrhage.

In conclusion, according to preliminary statistics and evaluation of 14 included studies, we concluded the following: NaoXueShu oral liquid in the treatment of acute cerebral hemorrhage had more obvious effects than conventional medical treatment (*P* < 0.01). To a certain extent, it can promote the absorption of hematoma in the brain and then rapidly reduce the brain hematoma volume (*P* < 0.05); however, Wang and Zhao [16] suggested that the absorption of intracranial hematoma did not represent an improvement in clinical outcome. Compared with the control group, there was no obvious advantage in the treatment of cerebral hemorrhage from 2 weeks with NIHSS (*P* > 0.05). However, compared with the NIHSS (*P* < 0.05) of 4 weeks and BI (*P* < 0.01) after 90 days, we found that NaoXueShu oral liquid can reduce the neurological damage and improve the prognosis. With NaoXueShu, drug taking time prolonged and the differences between the two groups increased gradually, which showed that long-term use can improve the neurological dysfunction of patients and their quality of life and living ability.

## Figures and Tables

**Table 1:** Characteristics of included studies.

Study	Sample size (treatment/control)	Diagnosis	Intervention		Treatment course	Clinical standards	Outcome measure
			Treatment	Control			
Y. Yuan and D. Yuan 2012 [23]	60 (36; 24)	CH	NaoXueShu + routine	Naoxuekang + Conventional treatment	30 days	The standard of TCM and Western medicine	Curative effect; hematoma absorption rate; integral value of neurological deficit; clinical symptom integral value of TCM
Wang et al. 2014 [24]	122 (64; 58)	HICH	NaoXueShu + routine	Conventional treatment	4 weeks	CH + standards (95)	GCS score; BI score; hematoma volume
Xie et al. 2007 [25]	440 (310; 130)	HS	NaoXueShu	Naoxuekang	30 days	TCM diagnostic code (96)	stroke score; hematoma uptake; therapeutic effect
Miao and Yan 2014 [26]	69 (35 : 34)	HICH	NaoXueShu + routine	Conventional treatment	4 weeks	CH standards (95) + CT	NIHSS score and curative effect
Lu et al. 2004 [27]	140 (70; 70)	HICH	NaoXueShu	Naoxuekang	1 month	Cranial CT	Changes in TCM syndrome score; NIH integral changes; intracranial hemorrhage
Wang and Zhao 2015 [16]	30 (15; 15)	ACH	NaoXueShu + routine	Conventional treatment	1 month	WHO diagnostics; CT; diagnostic criteria of stroke in Chinese Medicine	Hematoma uptake; stroke diagnosis score, NIHSS; 3-month mRS
Gu and Ma 2010 [28]	62 (32; 30)	CH	NaoXueShu + routine	Conventional treatment	1 month	CH standards (95)	Neurological deficit score; clinical efficacy
Chen et al. 2016 [17]	67 (35; 32)	HICH	NaoXueShu + routine	Conventional treatment	90 days	CT	Curative effect; hematoma volume change (10,20,25)
Wang and Ding 2014 (2) [29]	86 (43; 43)	CH	NaoXueShu + routine	Naoxuekang + Conventional treatment	1 month	Relevant standards of the Ministry of Health and criteria for diagnosis and treatment of stroke	Curative effect; hematoma volume change
Wang et al. 2016 [30]	96 (48; 48)	HICH	NaoXueShu + acupuncture + routine	Acupuncture + routine	1 month	Guidelines for prevention and treatment of cerebrovascular diseases in China (2005)	NIHSS score; BI score
Wang et al. 2015 (2) [18]	102 (51; 51)	Mixed stroke	NaoXueShu + routine	Conventional treatment	4 weeks	CT and/or MRI	WAB score; mRS score; NIHSS score; hemorheological indicators; (blood viscosity, fibrin, red blood cell index)
Zhou et al. 2015 [20]	170 (87; 83)	CH	NaoXueShu + routine	Conventional treatment	90 days	CT	ESS; Neurological deficit score; BI score
Li et al. 2014 [31]	76 (39; 37)	HICH	NaoXueShu + routine	Conventional treatment	2 weeks	CT	GCS score; Neurological deficit score
Yang and Xue 2015 [21]	86 (43; 43)	HICH	NaoXueShu + routine	Conventional treatment	4 weeks	CH standards (95)	NIHSS score; curative effect; hematoma

**Table 2:** Egger's test of publication bias.

Outcome	Std. Eff.	Coef.	Std. Err.	*t*	*P* > *t*	[95% CI]	
Hematoma volume	Slope	0.6955327	0.5746734	1.21	0.313	−1.133334	2.5244
	Bias	−7.826066	3.376481	−2.32	0.103	−18.57153	2.919403
NIHSS-2w	Slope	−0.3706095	0.6525846	−0.57	0.627	−3.178454	2.437235
	Bias	0.6525892	2.897941	0.23	0.843	−11.81624	13.12142
NIHSS-4w	Slope	2.208923	1.010601	2.19	0.094	−0.5969561	5.014802
	Bias	−13.91488	4.82455	−2.88	0.045	−27.30998	−0.5197859
Efficiency	Slope	−0.1368343	−0.0662528	−2.07	0.108	−0.3207815	−0.047113
	Bias	−0.1644329	0.244169	−0.67	0.538	−0.8423548	0.5134891

## Abbreviations

ACH: Acute cerebral hemorrhage
BI: Barthel Index
CNKI: Chinese National Knowledge Infrastructure
CBMdisc: Chinese Biomedical Literature Database
CT: Computer Tomography
CI: Confidence interval
CH: Cerebral hemorrhage
Coef.: Coefficient
GCS: Glasgow coma scores
HICH: Hypertensive intracerebral hematoma
HS: Hemorrhagic stroke
MD: Mean difference
mRS: Modified Rankin scale
NIHSS: National Institutes of Health Stroke Scale
OR: Odds ratio
RCTs: Randomized controlled trials
RR: Relative risk
Std. Eff.: Standard Effect
Std. Err.: Standard Error
TCM: Traditional Chinese Medicine
VIP: The Chinese Scientific Journal Database
WMD: Weighted mean difference
WHO: World Health Organization
WAB: Western aphasia battery
ESS: European Stroke Scale.
